# Metabolic reprogramming orchestrates an immunosuppressive microenvironment in anaplastic thyroid cancer: mechanisms and clinical perspectives

**DOI:** 10.3389/fimmu.2026.1699202

**Published:** 2026-02-04

**Authors:** Jinkun Xia, Baowei Zhai

**Affiliations:** Department of Vascular and Thyroid Surgery, Guizhou Provincial People’s Hospital, Guiyang, Guizhou, China

**Keywords:** anaplastic thyroid carcinoma, immune evasion, immune microenvironment, immunotherapy, metabolic reprogramming

## Abstract

Anaplastic Thyroid Carcinoma (ATC) represents one of the most aggressive and lethal human malignancies, characterized by rapid progression, profound therapy resistance, and a dismal prognosis. Recent advances have underscored metabolic reprogramming as a cornerstone of ATC pathogenesis, enabling tumor cells to adapt to a hostile microenvironment, sustain proliferation, and evade immune destruction. This review systematically delineates how metabolic alterations in ATC—spanning enhanced glycolysis, deregulated lipid metabolism, and aberrant amino acid utilization—orchestrate a profoundly immunosuppressive tumor immune microenvironment (TIME). We explore the mechanistic links between tumor metabolism and immune dysfunction, including nutrient competition-induced energy deficits in effector immune cells, accumulation of immunosuppressive metabolites, and metabolic regulation of immune checkpoint expression. Furthermore, we discuss the impact of metabolic crosstalk on immune cell phenotypes, fostering the recruitment and polarization of pro-tumorigenic immune populations such as M2 macrophages, regulatory T cells, and myeloid-derived suppressor cells. Clinically, we highlight the therapeutic promise of targeting key metabolic nodes and review emerging combination strategies that integrate metabolic inhibitors with immune checkpoint blockade to overcome resistance and enhance antitumor immunity. By synthesizing foundational insights with cutting-edge preclinical and clinical evidence, this review aims to provide a cohesive mechanistic framework and identify novel, metabolism-based therapeutic vulnerabilities for precision immunotherapy in ATC.

## 1 Introduction

Anaplastic thyroid carcinoma (ATC) is recognized as one of the most aggressive and lethal forms of thyroid cancer, characterized by rapid progression and poor prognosis. The survival duration for patients diagnosed with ATC is typically measured in months, marking it as a significant clinical challenge in oncology (1). This aggressive malignancy often arises from the dedifferentiation of well-differentiated thyroid tumors, such as papillary thyroid carcinoma (PTC), underscoring the need for a deeper understanding of its pathophysiology and the mechanisms driving its progression (2). Traditional treatment modalities, including surgery, chemotherapy, and radiotherapy, have shown limited efficacy (3). Clinical trials have revealed that immunotherapy elicits a superior response in ATC patients compared to those with advanced PTC, which is associated with lymphocyte infiltration. These infiltrating lymphocytes may promote the formation of early tertiary lymphoid structures (TLS) in ATC, thereby rendering it more sensitive to immunotherapy (4). Therefore, understanding the immune microenvironment of ATC may provide new treatment opportunities for patients with this malignant disease.

Metabolic reprogramming has emerged as a crucial characteristic of cancer cells, enabling them to adapt to the hostile tumor microenvironment (TME) and sustain rapid proliferation. This process involves significant alterations in various metabolic pathways, including glycolysis (the Warburg effect), lipid metabolism, and amino acid metabolism (5). In the context of thyroid cancer, metabolic reprogramming not only supports the survival and growth of tumor cells but also profoundly impacts the immune landscape, facilitating immune evasion and treatment resistance. The metabolic competition for nutrients, coupled with the accumulation of immunosuppressive metabolites like lactate and specific oncometabolites, directly impairs the function of effector immune cells while promoting the expansion and polarization of regulatory cell populations. Furthermore, metabolic reprogramming in cancer cells can upregulate immune checkpoint expression, such as PD-L1, creating a multi-faceted barrier to effective anti-tumor immunity (6).

The immune microenvironment plays a pivotal role in the development and progression of ATC (7). It is increasingly recognized that immune cells, including tumor-associated macrophages (TAMs) and T cells, are significantly influenced by the metabolic state of the TME (8). Metabolic alterations within tumor cells, such as increased glycolysis and altered lipid metabolism, can lead to the depletion of essential nutrients in the TME, thereby impairing the function of infiltrating immune cells (9). This dynamic interplay between tumor metabolism and immune cell function is critical for understanding the mechanisms of tumor immune evasion and developing effective immunotherapeutic strategies.

Recent advances in immunotherapy, particularly the use of immune checkpoint inhibitors, have shown promise in treating various malignancies, including ATC (10). However, the benefits of these therapies are often limited by the metabolic reprogramming of both tumor cells and immune cells, which can lead to adaptive resistance (11). Therefore, targeting metabolic pathways within tumor cells presents a novel approach to enhance the efficacy of immunotherapy and overcome resistance mechanisms. By restoring metabolic balance and improving the functional capacity of immune cells, these strategies have the potential to improve clinical outcomes for patients with ATC. While the individual roles of metabolic pathways in cancer cell survival are increasingly recognized, a comprehensive synthesis of how these pathways collectively orchestrate the immunosuppressive landscape in ATC is lacking. This review aims to bridge this gap by systematically elucidating the mechanisms through which metabolic reprogramming in ATC—specifically in glycolysis, lipid metabolism, and amino acid metabolism—coordinates the construction of an immunosuppressive TIME. Finally, we explore the compelling clinical prospects of targeting these metabolic pathways, both as standalone strategies and in rational combination with immunotherapy, to overcome treatment resistance and improve outcomes for ATC patients.

## 2 Metabolic reprogramming characteristics of anaplastic thyroid carcinoma

### 2.1 Enhancement of the glycolytic pathway and its therapeutic potential

Cancer cells are characterized by a pronounced reliance on aerobic glycolysis, commonly referred to as the Warburg effect, which enables them to meet the high energy demands associated with rapid proliferation (12). This metabolic shift allows cancer cells to convert glucose into lactate even in the presence of oxygen, a process that not only supports their growth but also contributes to the acidification of the tumor microenvironment, further promoting tumor progression and immune evasion (13). The conversion of pyruvate into lactate, catalyzed by lactate dehydrogenase (LDH), constitutes the final step of glycolysis. Inhibition of LDH has been demonstrated to suppress Warburg metabolism, leading to proapoptotic and antitumor effects in cancer cells. Once produced, lactate is exported out of the cell via monocarboxylate transporter 4 (MCT4) and functions as a signaling molecule and growth factor for adjacent tumor cells. Within tumor tissues, lactate secreted into the extracellular space through MCT4 can be taken up by neighboring cells via MCT1, a transporter that facilitates lactate influx. These cells subsequently utilize lactate as a metabolic substrate to support energy production and cellular growth. *In vitro* experiments show that inhibition of MCT4 with ACF markedly impairs ATC cell proliferation and glycolytic activity. Moreover, combined inhibition of MCT4 and MCT1—using either SYR or a combination of ACF and AZD—results in greater suppression of ATC cell growth compared to MCT1 inhibition alone (14). These findings support the potential of targeting glycolytic and lactate-processing pathways as an adjuvant treatment approach for ATC (14). Similarly, GLUT1, HK2, and LDHA are key glycolysis-associated genes involved in glucose uptake, glucose phosphorylation and intracellular retention, and the conversion of pyruvate to lactate, respectively. The inhibition of these genes by Se-methylselenocysteine, an organic selenium compound, has been demonstrated to block the progression of ATC (15).

Interestingly, epigenetic regulation, which represents a rapid and reversible response and involves diverse enzymes including histone (de)methylases, (de)acetylases, and DNA, has also been implicated in the glycolytic process of ATC (16). A recent study revealed that aerobic glycolysis in ATC cells increases the intracellular supply of lactate, thereby promoting global protein lactylation—a novel epigenetic modification (16). Moreover, the oncogenic BRAF V600E mutation enhances glycolytic flux, which in turn alters the cellular lactylation profile. Consequently, combined inhibition of lactylation mechanisms and BRAF V600E has been shown to effectively suppress ATC progression both *in vitro* and *in vivo* (16). These findings highlight an additional role of aerobic glycolysis in promoting ATC malignancy, unveiling a new metabolic–epigenetic regulatory axis and providing a rationale for combining BRAF V600E inhibitors with lactylation-targeting strategies in ATC therapy.

### 2.2 Remodeling of lipid metabolism in anaplastic thyroid carcinoma

In contrast to normal cells that primarily rely on dietary uptake for lipids, tumor cells predominantly utilize glucose metabolism as the source of raw materials for lipid synthesis (17). Notably, up to 93% of fatty acids in tumor cells are generated via *de novo* synthesis. Key enzymes involved in fatty acid biosynthesis—including ATP citrate lyase (ACLY), acetyl-CoA carboxylase (ACC), and fatty acid synthase (FASN)—are frequently altered in tumor cells. The process begins with citrate, an intermediate of glucose metabolism, which is converted into acetyl-CoA by ACLY. ACC then catalyzes the carboxylation of acetyl-CoA to form malonyl-CoA. Finally, FASN catalyzes the condensation of acetyl-CoA and malonyl-CoA to produce palmitic acid, which serves as a precursor for various cellular lipids through further modifications by other specific enzymes (18).

Recent investigation into the metabolic profile of ATC has revealed a tumor-specific dependency for increased *de novo* lipogenesis, offering new insight into the molecular mechanisms that govern disease initiation and progression (19). More than six decades ago, it was recognized that tumor tissues exhibit an increased demand for lipid biosynthesis, suggesting either enhanced uptake of lipids from the host or an acceleration of *de novo* lipogenesis to support tumorigenesis. In cancer cells, the upregulation of fatty acid transport proteins facilitates the uptake of free fatty acids from the extracellular environment (20). Recent studies have demonstrated the specific overexpression of various components involved in lipid biosynthesis across multiple cancer types. Moreover, tumor cells show susceptibility to targeted inhibition of these components, underscoring a specific reliance on lipid metabolism during oncogenesis. Tumors displaying a lipogenic phenotype are often associated with aggressive disease and poor patient prognosis (21).

Gene array analysis of ATC tissue with normal thyroid tissues revealed significant upregulation of enzymes promoting *de novo* fatty acid synthesis (22). These include ACC, FASN, as well as several fatty acid elongases and desaturases, notably the two human isoforms of stearoyl-CoA desaturase (SCD1 and SCD5). Additionally, increased expression was observed for proteins involved in fatty acid uptake, transport, and metabolism, including various fatty acid-binding proteins (FABPs) and solute carrier family 27 members (SLC27A) (23). Collectively, these alterations indicate an elevated demand for lipid bioavailability in this aggressive malignancy. A recent study revealed that ATC’s excessive uptake of fatty acids may be related to impaired autophagy function, leading to mitochondrial abnormalities and metabolic disorders - in which glucose is mainly used for glycolysis processes and therefore cannot be converted into fatty acids (19). As a result, the body’s lipid reserves gradually deplete, and in order to maintain important synthetic metabolic processes such as membrane synthesis, the body has to increase its intake of fatty acids.

In ATC, lipid metabolism is critically reprogrammed to meet the heightened demands for membrane synthesis and energy production necessary for rapid tumor growth and proliferation. ATC cells rely heavily on fatty acid synthesis and oxidation pathways to fulfill these metabolic needs. Consequently, the enhanced lipid synthesis and oxidation pathways allow ATC cells to thrive under these adverse conditions, promoting their survival and aggressive behavior. Furthermore, lipid metabolic products are increasingly recognized for their role in modulating immune responses within the tumor microenvironment (31). Tumor cells can reprogram cells within the tumor microenvironment (TME) toward a pro-tumor phenotype by secreting metabolites and signaling molecules—including cytokines and bioactive lipids. This communication promotes cancer progression through multiple mechanisms. For example, cancer-associated fibroblasts (CAFs), upon activation by tumor cells, upregulate lipid synthesis. These lipids not only supply energy to tumor cells but also contribute to an immunosuppressive microenvironment via the release of extracellular vesicles (EVs) and signaling factors. Similarly, immune cells in the TME undergo metabolic adaptations: tumor-infiltrating CD8+ T cells and natural killer (NK) cells show enhanced lipid uptake and fatty acid oxidation (FAO). Lipid accumulation in tumor-associated macrophages (TAMs) influences their polarization and functional state. Moreover, immunosuppressive cells such as regulatory T cells (Tregs) and myeloid-derived suppressor cells (MDSCs) also display increased lipid uptake and FAO, which reinforces their pro-tumor functions (22). This metabolic interplay not fuels tumor growth but also alters the functional phenotypes of TME components to support malignancy. By altering the lipid profiles within the tumor, ATC cells may evade immune surveillance and foster an environment conducive to tumor progression.

Among the upregulated lipogenic enzymes in ATC, SCD exhibited the most pronounced overexpression compared to normal thyroid tissue. SCD, a fatty acid desaturase, catalyzes the conversion of saturated fatty acids—produced by FASN activity—into monounsaturated fatty acids. Pharmacological or genetic inhibition of SCD activity effectively attenuates cell proliferation and induces cell death in ATC cells, while sparing normal thyroid cells (23). Interestingly, tumor cells derived from well-differentiated lesions exhibit resistance to SCD inhibition, suggesting a specific vulnerability in more aggressive thyroid malignancies.

Given the multifaceted roles of SCD activity and other neoplastic lipogenic components in cytoprotection, proliferation, migration, energy storage, and metabolism—processes that also contribute to chemoresistance—targeted inhibition of this pathway holds promise for combination with existing anticancer therapies to improve ATC treatment. In a preclinical study, combining the SCD inhibitor MF-438 with the proteasome inhibitor carfilzomib significantly suppressed ATC tumor growth (23). Furthermore, several successful examples support the efficacy of combination therapies targeting lipid metabolism in cancer models. For instance, co-administration of a FASN inhibitor with a thiazolidinedione (a PPARγ activator) demonstrated antitumor effects in prostate cancer cells (24), while in a preclinical model of colorectal cancer, the FASN inhibitor cerulenin synergized with oxaliplatin chemotherapy, yielding superior outcomes compared to either agent alone (25). Moreover, several studies targeting tumor lipid metabolism have also confirmed their potential therapeutic effects. For example, isoliquiritigenin (ISL), a bioactive isoflavonoid, could inhibit fatty acid synthesis by down-regulating the lipid synthesis-related enzyme expression level, and impede cancer cell migration in ATC through the AMPK/SREBF1 signaling pathway (26). Additionally, It’s reported that a combinational approach consisting of drugs designed for targeting lipid metabolism, lovastatin (an inhibitor of 3-hydroxy-3-methylglutaryl coenzyme A reductase, HMGCR) and troglitazone (an agonist of peroxisome proliferator-activated receptor gamma, PPARγ), exhibits anti-proliferation in cell culture systems and leads to tumor regression in ATC mouse xenograft model, suggesting that targeting two pathways involved in lipid metabolism may provide a new direction for treating ATC (27).

The heterogeneity of ATC and other aggressive malignancies poses a challenge of drug resistance following monotherapy. Although limited efficacy was observed with SCD inhibitor monotherapy in preclinical ATC models, significant synergistic effects emerged when combined with a proteasome inhibitor. Therefore, while the antitumor effects of individual lipid metabolism inhibitors warrant investigation, exploring their combination with radiotherapy, chemotherapy, and other targeted therapies represents a critical strategic direction.

### 2.3 Abnormalities in amino acid metabolism and their support for tumor growth

Glutamine is traditionally considered a non-essential amino acid in normal cells, as it can be synthesized from glucose. However, tumor cells exhibit a marked dependence on exogenous glutamine for survival in culture, effectively rendering it an essential amino acid in these contexts. Upon cellular uptake, glutamine is hydrolyzed by glutaminase into glutamate and ammonia. Glutamate can be further converted to α-ketoglutarate, which enters the tricarboxylic acid (TCA) cycle, thereby supplying intermediate metabolites and energy for cellular metabolism. Substantial evidence indicates that glutamine plays a critical role in supporting tumor growth. Cancer cells consume large quantities of glutamine, utilizing it as an important energy source complementary to glycolysis.

Multiple studies have highlighted alterations in glutamine metabolism within thyroid tumors. Inhibition of glutamine metabolism in thyroid cancer cells leads to energy deficiency, resulting in suppressed proliferation, migration, and invasion. In a tissue microarray analysis of 557 thyroid cancer cases, Kim et al. performed immunohistochemical staining for key glutaminolytic enzymes. Their findings revealed that glutaminase 1 (GLS1) and glutamate dehydrogenase (GDH) were expressed at the highest levels in thyroid cancer compared to other subtypes (28).

Amino acid metabolism plays a pivotal role in the growth and survival of cancer cells. One of the most critical aspects of this metabolic reprogramming is the enhanced metabolism of glutamine, an amino acid that serves multiple functions in tumor biology. Glutamine is not only a building block for protein synthesis but also a key substrate for nucleotide synthesis and a crucial player in maintaining cellular redox balance through its role in the synthesis of glutathione, a major antioxidant. Several studies showed the important roles of glutamine metabolism on ATC tumor environment. However, due to the complex crosstalk among glutamine metabolic pathways in ATC, other signaling pathways may exist that can bypass the stress response induced by glutamine inhibition. Yeseong Hwang and colleagues revealed that glutamine deprivation enhances ATF4-mediated one-carbon metabolism, and simultaneous suppression of both glutamine metabolism and one-carbon metabolism synergistically increased the anticancer efficacy of drugs used in ATC treatment (29).

Although the mechanisms of amino acid metabolism in ATC progression remain poorly characterized, the substantial energy demands associated with ATC’s aggressive growth and nutrient competition within TME suggest that amino acid metabolic reprogramming may support disease advancement. This adaptation presents a promising therapeutic vulnerability worth further investigation.

## 3 The impact of metabolic reprogramming on the immune microenvironment of anaplastic thyroid carcinoma

### 3.1 Metabolites regulate immune cell function

For a long time, lactate has been considered merely a waste product generated during cellular and tissue metabolism. However, research over the past decade has revealed the multifaceted functions of lactate, which have significant implications for cell biology. In addition to serving as an energy source, it has now been discovered that lactate can influence gene expression through histone modifications and act as a signaling molecule participating in various cellular activities. The two factors, lactate synthesis and lactate modification, jointly affect gene expression and protein function, acidify the tumor microenvironment, regulate immune evasion, and promote carcinogenesis (30). Within tumor cells, lactate can interfere with cellular signaling pathways, promote lactate transport, enhance cellular resistance to oxidative stress, and participate in lactylation reactions. Among various cell types, the interaction between lactate and immune cells regulates processes such as cell differentiation, immune response, immune surveillance, and therapeutic efficacy. In addition, the communication between lactate and stromal/endothelial cells also supports the remodeling of the basement membrane, epithelial mesenchymal transition, metabolic reprogramming, angiogenesis, and the formation of drug resistance. The research on the mechanism of lactate production and transport, especially through lactate dehydrogenase (LDH) and monocarboxylate transporter (MCT), has brought new hope for cancer treatment. Drugs that inhibit LDH and MCT can serve as both tumor inhibitors and enhance the efficacy of immunotherapy. When used in combination with immunotherapy, they can produce synergistic therapeutic effects (31).

In addition to lactate, lipid metabolism also has been proven to be a critical factor in regulating T cell responses. Specific lipid metabolites can act as “metabolic checkpoints,” helping T cells integrate external signals and interact with intracellular signaling processes. Extrinsically synthesized lipids and those present in the cell membrane play important roles in modulating T cell biology, exerting significant effects at the transcriptional, epigenetic, and post-translational regulatory levels (32). Similarly, lipid metabolism plays a pivotal role in regulating the immune responses of macrophages and dendritic cells. Metabolic dysregulations in various lipid metabolic pathways—including *de novo* fatty acid synthesis, fatty acid β-oxidation, lipid storage, and cholesterol efflux—can disrupt signaling mechanisms within immune cells, thereby leading to impaired function of both macrophages and dendritic cells in the tumor microenvironment (33).

Amino acid metabolism, particularly the catabolism of tryptophan through the indoleamine 2,3-dioxygenase (IDO) pathway, is another critical mechanism by which metabolites can regulate immune cell function (34). The IDO pathway’s activation not only hampers the effector functions of T cells but also skews the immune response towards tolerance, facilitating tumor progression and immune evasion (35). Understanding the mechanisms by which amino acid metabolism influences immune cell dynamics provides insights into potential therapeutic strategies aimed at counteracting the immunosuppressive effects of tumors, such as IDO inhibitors that could restore T cell function and enhance anti-tumor immunity.

The regulation of immune cell function by metabolic products, including lactate, lipid metabolites, and amino acid derivatives, underscores the intricate relationship between metabolism and immune responses in the tumor microenvironment. These metabolites not only shape the functional states of immune cells but also contribute to the immunosuppressive landscape that characterizes many tumors. Targeting these metabolic pathways presents a promising avenue for enhancing the effectiveness of cancer immunotherapy and improving patient outcomes.

### 3.2 Metabolic competition leads to energy deficiency in immune cells

**Figure 1:**
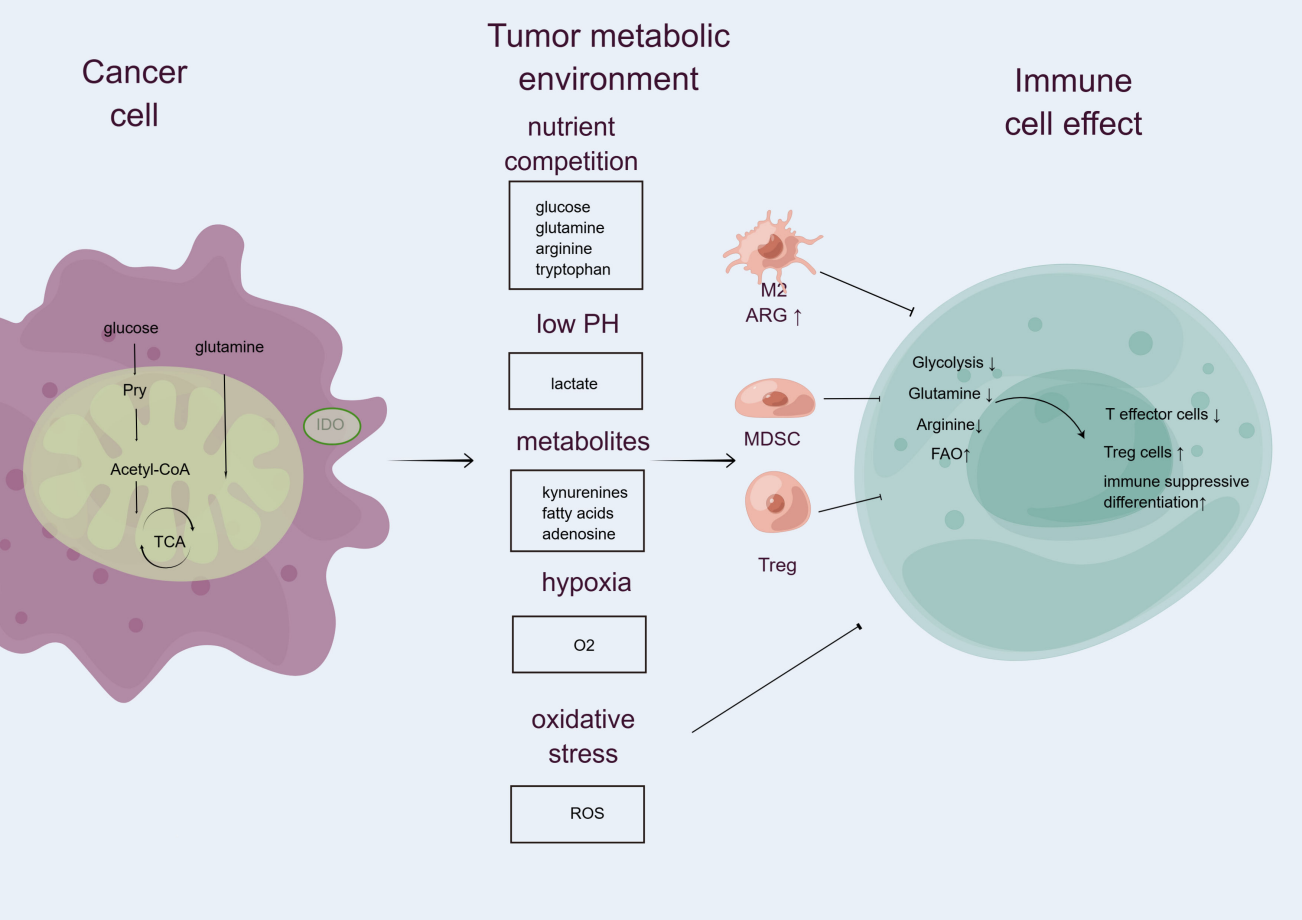
Competition for nutrient resources between immune and tumor cells within the tumor microenvironment critically influences immune cell activity (by FigDraw). Tumor cells, which often exhibit high metabolic rates, actively consume nutrients—including glucose, glutamine, arginine, and tryptophan—that are also essential for immune cell function. This metabolic rivalry leads to nutrient deprivation, impairing effector immune cell performance and limiting their clonal expansion. Furthermore, elevated glycolytic activity in tumor cells results in lactate accumulation, acidifying the TME and further suppressing effector immune responses, while enhancing the functions of immunosuppressive cells such as myeloid-derived suppressor cells (MDSCs), regulatory T cells (Tregs), and M2 macrophages. Additionally, tumor cells secrete various metabolites, including fatty acids, adenosine, and kynurenine, which promote the activation of immune-tolerant cell populations and concurrently inhibit antitumor effector functions. Hypoxia, driven by heightened oxygen consumption by tumor cells, along with the release of reactive oxygen species (ROS), further contributes to the suppression of anticancer immunity within the TME. M2, M2 macrophages; IDO, indoleamine 2,3-dioxygenase; ARG1, arginase 1; FAO, fatty acid oxidation; TCA, tricarboxylic acid; ROS, reactive oxygen species.

The metabolic landscape within the TME is characterized by fierce competition for limited nutrients, particularly glucose and amino acids, which are essential for both tumor cells and immune cells. Tumor cells, driven by their high proliferation rates, engage in metabolic reprogramming that allows them to efficiently utilize these nutrients, often at the expense of immune cell function (**Figure 1**). This phenomenon is particularly pronounced in aggressive cancers such as ATC, where the rapid consumption of glucose and amino acids by tumor cells creates a nutrient-deficient environment. As a result, immune cells, including T cells and macrophages, experience significant energy deficits, leading to impaired functionality (9). The reliance of immune cells on aerobic glycolysis for energy production means that when glucose is scarce, their ability to proliferate, secrete cytokines, and execute cytotoxic functions is severely compromised. Furthermore, the competition for amino acids, such as glutamine, exacerbates this issue, as tumor cells often outcompete immune cells for these critical substrates, further diminishing the immune response (9).

This metabolic competition manifests in various ways, including a shift in immune cell metabolism from oxidative phosphorylation to glycolysis, which is less efficient in terms of energy yield. For example, upon activation, immune cells reprogram their metabolic pathways to meet energy demands and biosynthetic requirements. Most lymphocytes, including inflammatory M1 macrophages, predominantly shift from oxidative phosphorylation to glycolysis, whereas regulatory T cells and M2 macrophages tend to rely more on the tricarboxylic acid (TCA) cycle and exhibit relatively low glycolytic activity (36). Additionally, the accumulation of metabolic byproducts, such as lactate, not only contributes to an acidic microenvironment but also directly inhibits T cell activation and proliferation, leading to a state of exhaustion. This state of immune cell dysfunction is further compounded by the presence of immunosuppressive cells within the TME, such as regulatory T cells and myeloid-derived suppressor cells (MDSCs), which themselves adapt their metabolism to thrive in low-nutrient conditions, thereby perpetuating the cycle of immune suppression (37).

The implications of these metabolic dynamics are profound, as they highlight the necessity for therapeutic strategies that can effectively modulate the metabolic competition within the TME. By targeting the metabolic pathways that tumor cells exploit, it may be possible to restore energy availability to immune cells, thereby enhancing their functionality and improving the efficacy of immunotherapies. For instance, interventions aimed at inhibiting key metabolic enzymes involved in glycolysis or glutaminolysis in tumor cells could alleviate the nutrient competition faced by immune cells, potentially reversing their energy deficits and restoring their capacity to mount effective anti-tumor responses (38). Moreover, the development of combination therapies that integrate metabolic modulators with existing immunotherapies could represent a promising avenue for enhancing treatment outcomes in patients with malignancies characterized by metabolic reprogramming (39). Ultimately, understanding and addressing the metabolic competition in the TME is crucial for devising innovative strategies to combat tumor-induced immune evasion and improve patient outcomes in cancer therapy.

### 3.3 Metabolic regulation of immunosuppressive factor expression

Metabolic reprogramming within the TME significantly influences the expression of immunosuppressive factors, such as immune checkpoint molecules, which are crucial for tumor immune evasion. One of the prominent mechanisms by which metabolic changes promote immunosuppression involves the upregulation of programmed death-ligand 1 (PD-L1) and cytotoxic T-lymphocyte-associated protein 4 (CTLA-4) on tumor cells and immune cells (40). The expression of these immune checkpoints is often linked to the metabolic state of the tumor, particularly the reliance on glycolysis and glutaminolysis. For instance, studies have shown that high levels of lactate can enhance PD-L1 expression through transcriptional activation by hypoxia-inducible factor 1-alpha (HIF-1α) (41). This metabolic shift not only supports tumor growth but also creates an immunosuppressive environment by inhibiting T-cell activation and function. Additionally, the accumulation of certain metabolites, such as succinate and fumarate, can stabilize HIF-1α, further driving the expression of PD-L1 and other immunosuppressive factors. This interplay between metabolism and immune checkpoint expression highlights the critical role of metabolic pathways in mediating immune evasion in tumors.

## 4 Metabolic reprogramming and immune evasion mechanisms in thyroid undifferentiated cancer

The phenomenon of T cell exhaustion is a significant barrier to effective cancer immunotherapy, particularly in the context of metabolic stress within the TME. Prolonged metabolic pressure can lead to the exhaustion of effector T cells, which is characterized by high expression levels of PD-1 and a subsequent loss of functionality (42). This state of exhaustion is not merely a passive decline in T cell activity; rather, it reflects a complex interplay of metabolic dysregulation, altered signaling pathways, and epigenetic modifications that collectively impair T cell responses to tumor antigens (42). For instance, terminally exhausted CD8+ tumor-infiltrating lymphocytes (TILs) exhibit a diminished capacity for cytokine production and proliferative responses, which are essential for effective anti-tumor immunity. Recent studies have demonstrated that metabolic reprogramming, particularly through oxidative phosphorylation, can reinvigorate exhausted T cells, enhancing their effector functions and improving responses to immunotherapy (43). This metabolic shift is crucial, as it underscores the potential for therapeutic strategies aimed at restoring metabolic fitness in T cells to combat exhaustion and enhance anti-tumor immunity.

Moreover, metabolic abnormalities within the TME can induce significant phenotypic changes in immune cells, further diminishing anti-tumor immune efficacy. For example, the metabolic reprogramming of T cells often results in a reliance on glycolysis over oxidative phosphorylation, which can lead to an impaired capacity for sustained effector function and survival (44). In various cancer types, such as clear cell renal cell carcinoma (ccRCC), distinct metabolic subtypes have been identified, with specific metabolic pathways correlating with immune exhaustion markers. The MT2 metabolic subtype, for instance, is associated with a poor prognosis and a reduced response to immunotherapy, highlighting the detrimental effects of abnormal metabolism on T cell functionality (45). This relationship emphasizes the importance of understanding the metabolic landscape of tumors, as it not only affects tumor growth but also shapes the immune response, leading to a more immunosuppressive environment that favors tumor progression.

In addition to glycolytic shifts, the accumulation of specific metabolites, such as lipids, can further exacerbate immune cell exhaustion (46). This metabolic dysfunction is often coupled with an increase in Tregs and TAMs, which can create a feedback loop that perpetuates immune suppression and T cell exhaustion. The interplay between these immune cell populations and their metabolic states is critical for understanding how to effectively target the TME to restore T cell function and enhance the overall efficacy of cancer immunotherapy.

Moreover, the regulation of immune cell infiltration and function is significantly influenced by metabolic products derived from tumor cells. For instance, the accumulation of certain metabolites such as glutamine and fatty acids can alter the functional state of immune cells within the tumor microenvironment (TME). Tumor cells often exhibit a high demand for these nutrients, leading to competitive inhibition of immune cell metabolism and function. This competition can result in a diminished capacity of immune cells, particularly T cells, to mount effective anti-tumor responses. Additionally, the metabolic reprogramming of immune cells themselves, such as the shift towards aerobic glycolysis, can further complicate the immune landscape. While this shift may initially enhance the effector functions of T cells, prolonged exposure to the altered metabolic environment can lead to T cell exhaustion and dysfunction, characterized by the upregulation of inhibitory receptors like PD-1. The complex interactions between tumor metabolism and immune cell function highlight the necessity of understanding these metabolic pathways to develop effective therapeutic strategies.

The mechanisms underlying metabolic-induced immune cell exhaustion are multifaceted and involve a combination of metabolic reprogramming, altered signaling pathways, and the accumulation of immunosuppressive metabolites. Addressing these metabolic challenges through targeted interventions may provide novel therapeutic avenues to rejuvenate exhausted T cells and enhance their anti-tumor responses, ultimately improving clinical outcomes for cancer patients undergoing immunotherapy. As research continues to unravel the complexities of T cell metabolism in the context of cancer, it becomes increasingly clear that a comprehensive understanding of these processes is essential for developing effective strategies to combat T cell exhaustion and enhance anti-tumor immunity (47).

## 5 Immune therapy strategies for anaplastic thyroid carcinoma based on metabolic reprogramming

### 5.1 Development and application of metabolic target drugs

The development of small molecule inhibitors targeting key glycolytic enzymes or critical metabolic process has shown promising antitumor effects in ATC models. The isozyme 6-phosphofructo-2-kinase/fructose-2,6-bisphosphatase 3 (PFKFB3) plays a critical role in promoting glycolysis, proliferation, and metastasis in various cancers, and the small-molecule inhibitor KAN0438757 was introduced as a potent and specific PFKFB3 antagonist, demonstrating efficacy in suppressing colon cancer growth (48). PFKFB3 could enable ATC cells to survive under hypoxic conditions by enhancing glycolytic flux, which leads to lactic acid production and subsequent acidification of the tumor microenvironment through the AKT–WNT/β-catenin signaling pathway, thereby facilitating invasion and metastasis; thus, PFKFB3 antagonist can also be considered as a potential drug for treating ATC (49). Additionally, phytochemicals, which are bioactive components derived from plants, can alter signaling cascades and modulate the metabolic properties of ATC cells, thereby highlighting their potential as alternative therapies to prevent drug resistance (50, 51). Furthermore, study revealed that during prolonged exposure to doxorubicin, ATC cells exhibited a time-dependent significant increase in 6-phosphogluconate dehydrogenase (6PGD) mRNA and protein levels, as well as enzymatic activity. Genetic and pharmacological inhibition of 6PGD effectively suppressed the growth and survival of these highly doxorubicin-resistant cells. This inhibition also enhanced the sensitivity of ATC cells to doxorubicin treatment. These findings indicate that suppressing 6PGD disrupts the metabolic reprogramming of doxorubicin-resistant ATC cells, underscoring the critical role of metabolic reprogramming in chemotherapy resistance in this cancer type (52).

In addition to glycolytic inhibitors, lipid metabolism inhibitors, such as fatty acid synthase (FASN) inhibitors, have demonstrated the ability to enhance immune responses when combined with immune checkpoint inhibitors (53). FASN is instrumental in *de novo* lipogenesis, a process that supports tumor growth and immune evasion by providing lipids for membrane synthesis and signaling. By inhibiting FASN, the metabolic landscape of the tumor is altered, leading to decreased lipid availability that can enhance the effectiveness of immune checkpoint therapies (54). This combination approach not only targets the metabolic vulnerabilities of ATC but also reinvigorates antitumor immunity, suggesting a synergistic effect that could improve clinical outcomes for patients with this aggressive cancer type. The integration of metabolic targeting with immunotherapy represents a novel and promising avenue for enhancing treatment efficacy in ATC.

### 5.2 Metabolic regulation and combination therapy with immune checkpoint inhibitors

Metabolic regulation plays a crucial role in enhancing the efficacy of immune checkpoint inhibitors (ICIs) in cancer therapy (55). By targeting metabolic pathways, researchers aim to improve the metabolic state of immune cells, thereby enhancing their functionality and responsiveness to ICIs. For instance, the metabolic reprogramming of T cells can be achieved through various strategies, such as inhibiting glycolysis or modulating lipid metabolism. These interventions can lead to an increase in the cytotoxic potential of T cells and a reduction in the immunosuppressive effects of the TME, ultimately improving the therapeutic outcomes of ICIs like PD-1 and PD-L1 inhibitors (56). Moreover, the combination of metabolic regulators with ICIs has shown promising results in preclinical models, where metabolic inhibitors not only restore T cell function but also enhance the overall antitumor immune response. This synergistic approach is particularly relevant in cancers that exhibit resistance to standard ICI therapies due to metabolic adaptations (57).

Clinical trials have begun to explore the combination of metabolic regulators with PD-1/PD-L1 antibodies, yielding preliminary data that support the potential of this strategy. For example, studies have demonstrated that the use of monocarboxylate transporter (MCT) inhibitors in conjunction with immune checkpoint blockade can significantly enhance T cell responses and improve tumor control (58). In these trials, patients receiving the combination therapy exhibited increased T cell infiltration and a more favorable immune profile compared to those treated with ICIs alone. Additionally, the combination of dihydroorotate dehydrogenase (DHODH) inhibitors with dual immune checkpoint blockade (anti-CTLA-4 plus anti-PD-1) has shown to upregulate antigen presentation pathways in tumor cells, thereby enhancing their visibility to the immune system and improving patient survival outcomes (59). These findings underscore the importance of integrating metabolic modulation into the treatment regimens of patients receiving ICIs, as they may help overcome the limitations posed by the TME and improve the overall efficacy of immunotherapy.

Furthermore, the exploration of specific metabolic pathways has revealed novel biomarkers that could predict the response to combination therapies involving ICIs and metabolic regulators. For instance, the expression of certain metabolic genes has been correlated with immune cell infiltration and the expression of immune checkpoints in tumors, suggesting that these markers could serve as valuable tools for patient stratification in future clinical trials (60). The ongoing research into the interplay between metabolism and immune regulation continues to highlight the potential for developing more effective combination therapies that can address the challenges of immune resistance in cancer treatment.

### 5.3 Metabolic regulators in immune cell therapy potential

The optimization of chimeric antigen receptor T (CAR-T) cells and tumor-infiltrating lymphocytes (TILs) through metabolic reprogramming has emerged as a promising strategy to enhance their anti-tumor efficacy. By understanding and manipulating the metabolic pathways that govern T cell function, researchers have begun to identify ways to improve the metabolic fitness of CAR-T cells and TILs, thereby enhancing their proliferation, survival, and effector functions in the TME. For instance, studies have shown that CAR-T cells can be engineered to utilize alternative metabolic pathways, such as fatty acid oxidation or oxidative phosphorylation, which may confer a survival advantage in the nutrient-poor environment typical of solid tumors (61). Furthermore, the modulation of glucose metabolism in T cells has been linked to their activation and differentiation states. Enhanced glycolysis has been associated with improved T cell activation, while oxidative phosphorylation is critical for memory T cell formation. Therefore, targeting these metabolic pathways can potentially lead to the development of CAR-T cells with superior anti-tumor activity, capable of overcoming the metabolic challenges posed by the TME.

Recent advancements in the field of metabolic regulators have also highlighted their role in supporting the expansion and functional maintenance of immune cells during therapy. For instance, the use of metabolic modulators such as 2-deoxyglucose, which inhibits glycolysis, has been shown to enhance the anti-tumor functions of TILs by promoting their activation and reducing exhaustion markers (62). Additionally, the incorporation of specific amino acids, such as arginine and glutamine, into culture media for T cell expansion has been demonstrated to improve T cell proliferation and functionality. These amino acids serve not only as building blocks for protein synthesis but also as critical regulators of T cell metabolism and signaling pathways (63, 64). Moreover, the identification of metabolic checkpoints, such as those mediated by the mechanistic target of rapamycin (mTOR) pathway, has provided new insights into how metabolic interventions can be employed to enhance T cell responses. By strategically targeting these metabolic pathways, it is possible to create a more favorable metabolic environment for T cells, thereby improving their therapeutic potential in cancer immunotherapy (65).

### 5.4 Challenges in clinical translation of metabolic-targeting drugs combined with immunotherapy

One major challenge of the combination of metabolic-targeting drugs with immunotherapy is the off-target toxicity associated with metabolic-targeting drugs. As highlighted by (66), these drugs often exhibit unintended effects on effector immune cells, such as T cells, due to the shared metabolic pathways between tumor cells and immune cells. For instance, inhibitors targeting glycolysis or oxidative phosphorylation can impair the function and proliferation of cytotoxic T lymphocytes, leading to compromised immune responses and increased susceptibility to infections or autoimmune side effects. This toxicity is exacerbated by the high dosages required for efficacy, resulting in systemic side effects that limit patient tolerance and treatment adherence. Nanotechnology offers a potential solution by enabling targeted delivery to tumor sites, thereby minimizing off-target effects and reducing required doses. Despite this, the clinical implementation of such nano-formulations requires rigorous safety profiling to avoid novel toxicities from carrier materials.

Another critical challenge is the development of resistance, driven by the metabolic heterogeneity of tumor-infiltrating T cells. Peipei Li et al. emphasizes that T cells within the TME display diverse metabolic states across cancer types, influenced by factors like nutrient deprivation and hypoxia (67). This heterogeneity means that metabolic-targeting drugs may only affect specific T cell subpopulations, allowing resistant clones to proliferate and evade therapy. For example, drugs targeting PD-1 or CTLA-4 in combination with metabolic modulators could fail in patients with T cells that exhibit alternative metabolic addictions, such as fatty acid oxidation. This resistance mirrors the dynamics seen in targeted therapies, where tumor evolution leads to adaptive metabolic bypass mechanisms. Single-cell sequencing technologies could identify resistant metabolic signatures early, enabling personalized combination regimens. However, the clinical adoption of such approaches is hindered by cost and technical complexities.

From a novel perspective, the integration of metabolic profiling with real-time monitoring could mitigate these challenges. For instance, clinicians could stratify patients based on T cell metabolic diversity using biomarkers from liquid biopsies, tailoring drug combinations to individual metabolic vulnerabilities. Additionally, nanotechnology suggests that smart nanocarriers with stimuli-responsive release could dynamically adjust drug delivery in response to TME changes, reducing toxicity and overcoming resistance by targeting multiple pathways simultaneously. This approach aligns with the trend towards precision immunometabolism but requires validation in large-scale trials to establish efficacy and safety.

## 6 Challenges and future directions in metabolic reprogramming research

### 6.1 Metabolic heterogeneity and its impact on treatment response

Metabolic heterogeneity in ATC poses significant challenges in treatment response, complicating therapeutic strategies. ATC is characterized by its aggressive nature and poor prognosis, often exhibiting a wide range of metabolic profiles among tumor cells. This heterogeneity arises from various factors, including genetic mutations, microenvironmental influences, and the adaptive responses of cancer cells to therapeutic pressures. For instance, cancer cells can switch between different metabolic pathways, such as glycolysis and oxidative phosphorylation, depending on the availability of nutrients and oxygen levels within the tumor microenvironment. This metabolic plasticity allows tumor cells to survive and proliferate even in the face of targeted therapies, leading to treatment resistance (68, 69). Furthermore, the presence of different metabolic subtypes within ATC can result in varying responses to treatments, such as chemotherapy and immunotherapy. For example, tumors with high glycolytic activity may respond differently to agents targeting oxidative phosphorylation compared to those with a more oxidative phenotype. This underscores the necessity for a deeper understanding of the metabolic landscape within ATC tumors to enhance treatment efficacy.

To address the challenges posed by metabolic heterogeneity, precise metabolic phenotyping is essential for guiding personalized treatment approaches. Recent advancements in imaging techniques, such as positron emission tomography (PET) and magnetic resonance spectroscopy (MRS), enable the assessment of metabolic activity *in vivo*, providing insights into the metabolic profiles of individual tumors (70, 71). Such technologies can help identify specific metabolic vulnerabilities that can be targeted therapeutically. For instance, tumors characterized by elevated levels of certain metabolites may be more susceptible to therapies that disrupt their metabolic pathways. Additionally, integrating metabolic profiling with genomic and transcriptomic analyses can further refine patient stratification and treatment selection, ensuring that patients receive the most effective therapies tailored to their tumor’s unique metabolic characteristics (72, 73).

Moreover, understanding the interplay between tumor metabolism and the immune microenvironment is crucial in the context of immunotherapy. Metabolic reprogramming in tumor cells can lead to the secretion of immunosuppressive factors that hinder effective immune responses (74). Consequently, therapies that target both metabolic pathways and immune modulation may enhance treatment responses in ATC patients. Overall, addressing metabolic heterogeneity through precise phenotyping and personalized treatment strategies holds promise for improving outcomes in patients with ATC. By leveraging the insights gained from metabolic analyses, clinicians can develop more effective, individualized treatment regimens that account for the unique metabolic landscape of each tumor, ultimately enhancing therapeutic efficacy and patient survival.

### 6.2 Metabolic reprogramming and the complex interactions of the immune microenvironment: systems biology research

The intricate relationship between metabolic reprogramming and the immune microenvironment has emerged as a focal point in cancer research, highlighting the dynamic interplay that shapes tumor progression and therapeutic responses. Recent advancements in single-cell sequencing and spatial omics technologies have revolutionized our understanding of these interactions, allowing for the dissection of metabolic and immune cell dynamics within the TME. These cutting-edge techniques enable researchers to map the metabolic states of individual cells and their spatial organization within tumors, revealing how metabolic alterations influence immune cell function and vice versa. For instance, studies utilizing single-cell RNA sequencing have demonstrated that metabolic reprogramming in cancer cells can lead to the secretion of metabolites that modulate immune cell behavior, promoting an immunosuppressive environment conducive to tumor growth (75). Additionally, spatial transcriptomics has uncovered distinct metabolic signatures associated with various immune cell populations, elucidating how these cells adapt their metabolism in response to the TME’s nutrient availability and stressors (76). The integration of these technologies facilitates the construction of comprehensive metabolic-immune regulatory networks, which can inform the design of precision therapies that target specific metabolic pathways to enhance anti-tumor immunity.

Moreover, the development of metabolic-immune regulatory network models offers a promising avenue for guiding therapeutic interventions. By mapping the interactions between metabolic pathways and immune cell functions, researchers can identify critical nodes within these networks that may serve as potential therapeutic targets. For example, targeting specific metabolic enzymes that are upregulated in TAMs has shown promise in reprogramming these cells from a pro-tumorigenic to an anti-tumor phenotype, thereby enhancing the overall immune response against tumors (77). Furthermore, the establishment of systems biology frameworks allows for the simulation of metabolic-immune interactions, providing insights into how perturbations in metabolic pathways can influence immune cell activation and differentiation. This approach not only aids in understanding the underlying mechanisms of immune evasion but also helps in predicting the outcomes of various therapeutic strategies, thereby paving the way for more effective cancer treatments.

In conclusion, the application of single-cell sequencing and spatial omics technologies is crucial in unraveling the complex interactions between metabolic reprogramming and the immune microenvironment in cancer. By constructing detailed metabolic-immune regulatory network models, researchers can identify novel therapeutic targets and design precision therapies that leverage the metabolic vulnerabilities of tumors and their associated immune cells.

## 7 Conclusion

In recent years, the understanding of ATC has evolved significantly, particularly concerning the role of metabolic reprogramming as a critical mechanism by which ATC cells adapt to their microenvironment and evade immune surveillance. This review has highlighted the intricate relationship between metabolic reprogramming and the immune landscape of ATC, underscoring the importance of this phenomenon in the progression and treatment of the disease.

Metabolic reprogramming is not merely a byproduct of tumorigenesis; it is a fundamental strategy employed by ATC cells to thrive under adverse conditions. By altering their metabolic pathways, these cancer cells can manipulate the immune microenvironment to their advantage. The regulation of immune cell metabolism and the expression of immunosuppressive factors are pivotal in shaping the tumor’s immune landscape, leading to a more favorable environment for tumor growth and progression. This dynamic interplay between tumor metabolism and immune response presents both challenges and opportunities in the treatment of ATC.

The advent of metabolism-based immunotherapeutic strategies represents a promising frontier in ATC management. The potential for combining metabolic-targeting agents with immune checkpoint inhibitors offers a novel approach that could enhance therapeutic efficacy. Such combinations may not only mitigate the immunosuppressive effects of the tumor microenvironment but also reinvigorate the immune response against ATC cells. The encouraging results from preliminary studies indicate that this dual approach could significantly improve patient outcomes, paving the way for more effective treatment regimens.

It is imperative to deepen our understanding of metabolic heterogeneity within ATC and its implications for the immune microenvironment. This necessitates a concerted effort to identify and validate metabolic biomarkers that could be utilized in clinical settings, providing insights into patient stratification and treatment response. Furthermore, advancing the field of systems biology to elucidate the complex mechanisms underlying metabolic reprogramming will be essential for translating these findings into clinical practice. In conclusion, the intersection of metabolism and immunology in the context of ATC presents a rich landscape for future research and therapeutic innovation. Balancing the diverse research perspectives on metabolic reprogramming and immune evasion will be crucial in developing comprehensive treatment strategies.
